# Shear Strength Range of GF/Polyester Composites Controlled by Plasma Nanotechnology

**DOI:** 10.3390/polym15163331

**Published:** 2023-08-08

**Authors:** Veronika Sirjovova, Milan Zvonek, Michal Jurko, Vladimir Cech

**Affiliations:** Institute of Materials Chemistry, Faculty of Chemistry, Brno University of Technology, Purkynova 118, 61200 Brno, Czech Republic

**Keywords:** glass fibers, polymer-matrix composites, shear strength, plasma nanotechnology

## Abstract

Unsized single-end rovings are oxygen plasma pretreated and organosilicon plasma coated using plasma nanotechnology to optimize the interphase in glass-fiber-reinforced polyester composites and to determine the achievable range of their shear strength for potential applications. This surface modification of the fibers allows us to vary the shear strength of the composite in the range of 23.1 to 45.2 MPa at reduced financial costs of the process, while the commercial sizing corresponds to 39.2 MPa. The shear strength variability is controlled by the adhesion of the interlayer (plasma nanocoating) due to the variable density of chemical bonds at the interlayer/glass interface. The optimized technological conditions can be used for continuous surface modification of rovings in commercial online fiber-processing systems.

## 1. Introduction

Today, plasma treatment and plasma coating belong to progressive techniques of fiber surface modification that are used as reinforcements in polymer composites [1]. The purpose of these modifications is to improve adhesion at the interface between the fiber and the polymer matrix to ensure efficient stress transfer from the matrix to the fiber when the composite is subjected to mechanical or thermal loading. This adhesion at the fiber/matrix interface is responsible not only for the shear properties of the fiber-reinforced composite (FRC), but also partly for its flexural, tensile, and compressive properties, thus determining the FRC performance during its service life in both indoor and outdoor environments [2,3]. Plasma processing can be used for both fibers and nanofibers [4]. Many studies using nonthermal plasma, characterized by low kinetic energy of neutral and ionic species (≈300 K), are devoted to carbon fibers [5,6], glass [7,8], aramid [9], polyethylene [10] or natural fibers [11,12,13]. In the case of a thermoplastic matrix, the fibers are plasma treated in argon, oxygen, nitrogen, ammonia or in their mixtures, and in the air in order to ablate or etch the smooth surface of the fibers and thereby increase the roughness of the surface, or to oxidize (air, oxygen plasma) the surface of the fiber to improve its wettability, both of which lead to improved interfacial adhesion [14]. However, this approach must be optimized because it can cause a decrease in fiber tensile strength not only for natural fibers [10,12], but also for compact and strong aramid [9] or carbon fibers [15], and, in principle, the improved interfacial adhesion is relatively low, as it uses only mechanical keying and weak van der Waals forces. In the case of a thermosetting matrix, the plasma treatment must be supplemented by plasma coating, with a functional interlayer inserted between the fiber and the matrix to significantly increase the interfacial adhesion due to the strong chemical bonding at both interfaces [16]. Plasma treatment of commercially sized fibers leads to the degradation of functional groups in this sizing, reducing interfacial adhesion [17]. Atmospheric and low-pressure plasmas [5,18] can be used both in batch processing systems and in online processing systems using differential pumping in the case of low pressure.

Plasma nanotechnology makes it possible to deposit functional interlayers that control the shear properties of fiber-reinforced composites with thermosetting resin, as demonstrated by previous studies [19,20]. A bundle of fibers was uniformly coated in a tubular plasma reactor and the adhesion of the interlayer (plasma coating) and its chemical properties were approximately the same for all fibers in the bundle as found by Zvonek et al. [19]. Plichta et al. then showed the correlation between the shear strength of the composite reinforced with plasma-coated fibers and interlayer adhesion [20]. This paper focuses on glass fibers (GFs) designed to reinforce polyester resin that were pretreated with oxygen plasma and subsequently plasma coated using tetravinylsilane (TVS) precursor, pure or mixed with oxygen gas. Oxygen plasma pretreatment removes the adsorbed gas from the GF surface and allows reproducible adhesion of the plasma coatings, and the mixture of oxygen with the precursor was found to be beneficial to the shear strength of the composite in an initial study using only a small laboratory plasma device [21]. The TVS molecule is dissociated by high-energy electrons in the nonthermal plasma, so the silicon-containing plasma species (radicals) are expected to be chemisorbed on the GF surface, and the vinyl group is important for chemical bonding with the polyester resin during the curing process. The effect of the discharge power and the oxygen fraction in the mixture on the short-beam strength of the composites was investigated in this study. The goal was to determine the achievable range of shear strength for potential applications and to understand the interlayer adhesion with respect to the density and type of chemical bonding. The shear strength requirements of a composite product are quite different, and therefore, the range of shear strength offered should be as wide as possible.

## 2. Materials and Methods

A single-segment reactor [22] using nonthermal radio frequency plasma (RF, 13.56 MHz) was used for plasma pretreatment and the plasma coating of fibers or silicon wafers in the continuous wave mode. The unsized single-end roving (1200 tex, Saint-Gobain Adfors, Litomysl, Czechia) or silicon wafer (<100>, ON Semiconductor, Roznov pod Radhostem, Czechia) was pretreated with oxygen plasma (10.0 sccm, 5.0 Pa) at an RF power of 100 W for 30 min; the base pressure was 5 × 10^−4^ Pa. Pure TVS precursor (Si–(CH=CH_2_)_4_, Sigma Aldrich, Praha, Czechia) or mixed with oxygen gas (Linde Gas, Brno, Czechia) with an oxygen fraction from zero to 0.71 used for plasma coating of substrates at RF power from zero to 200 W and a total flow rate of 6.0 sccm. The oxygen fraction is calculated as the ratio of the oxygen flow rate and the total oxygen and precursor flow rate. Since the oxygen flow rate meter starts at 2.0 sccm, the corresponding oxygen fraction is 0.33, and the other oxygen fractions are 0.42, 0.52, 0.61 and 0.71.

Short beams (18 × 10 × 3 mm^3^) of unidirectional GF/polyester composites with a fiber volume fracture of 39% were hand fabricated [2] and cured for 30 min at 100 °C and then for 1 h at 140 °C, using an unsaturated polyester resin Distitron 183 B1 [23] (Polynt S.p.A., Scanzorosciate, Italy). Eight beams from each coating type were tested in three-point bending using a Materials Testing Machine (AllroundLine Z010 TE, ZwickRoell, Ulm, Germany) according to ASTM D2344 [24]. The ratio of span length to specimen thickness was 4 and the test speed was set to a crosshead movement of 1 mm min^−1^.

The work of adhesion [20] for 0.1 µm thick plasma nanocoatings deposited on silicon wafers was determined based on a nanoscratch test using a 2D TriboScope-75 (Hysitron, Minneapolis, MN, USA) attached to a NTEGRA Prima Scanning Probe Microscope (NT-MDT, Zelenograd, Russia). A conical diamond indenter (Hysitron, Minneapolis, MN, USA) with a tip radius of 1 µm was used to create 10 μm scratches in 30 s under a linearly increasing normal load from 2 µN to 6 mN.

Fourier transform infrared (FTIR) transmission spectra of plasma nanocoatings on IR transparent silicon wafers were recorded with a VERTEX 80v spectrometer (Bruker Optics, Billerica, MA, USA) in the range 4000–500 cm^−1^ using 256 scans and a spectral resolution of 4 cm^−1^.

## 3. Results and Discussion

**Figure 1:**
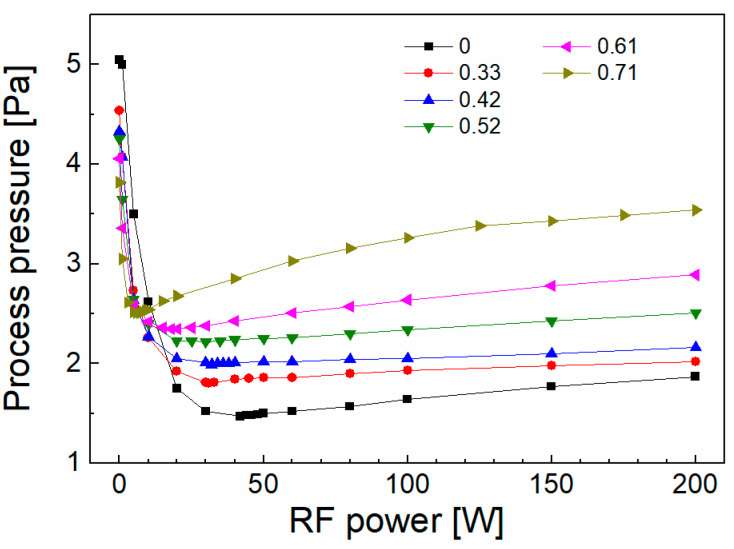
Power-dependent process pressure for different amounts of oxygen in the TVS/O_2_ mixture.

Plasma nanotechnology uses nonthermal plasma with a higher degree of dissociation, which results in a more efficient use of the precursor molecules and thus a reduction in the financial costs of the process [25]. The drop in process pressure is characteristic of a nonthermal plasma with a higher degree of dissociation, because of the fast chemisorption of fragmented (dissociated) precursor molecules on the surface of the growing coating. The process pressure as a function of the RF power for different oxygen fractions is shown in Figure 1. Typically, the initial pressure at zero power decreased to a minimum, where chemisorption is the most efficient, followed by a partial increase with enhanced power.

**Figure 2:**
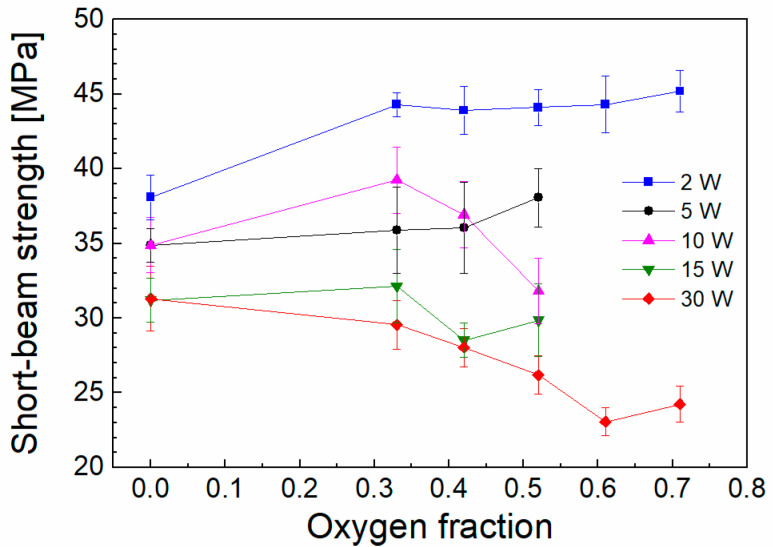
Range of short-beam strength as a function of oxygen fraction for different powers.

The effectivity of plasma surface modification of fibers in a bundle or woven and non-woven fabrics depends on the process pressure used. The mean free path of plasma species increases with decreasing pressure [26] and only in the case of low-pressure plasma the plasma species can penetrate the bundle or fabrics, but their activity is affected by the shielding effect of surrounding fibers. Plasma species cannot penetrate effectively into compact fabrics [6,7,8,10,15], and therefore, plasma treatment and plasma coating are effective only to a certain depth below the outer surface of the fabrics. The deposition time (68–1020 s) was calculated [22] based on knowledge of the number of filaments in the bundle (1600), the shielding factor (0.9), and the deposition rate (13–171 nm min^−1^) for a given power and oxygen fraction to cover even the central filament in a bundle with a nanocoating of at least 20 nm thickness. The short-beam strength of composites reinforced with fibers that were plasma-coated from a TVS precursor mixture with oxygen gas (oxygen fraction from zero to 0.71) at different RF power (2–30 W) is given in Figure 2. The highest shear strength range of 23.1 to 45.2 MPa, corresponding to an oxygen fraction of 0.61–0.71, decreased to 31.3–38.1 MPa for the zero-oxygen fraction and will be discussed in the next paragraphs based on the assessment of interlayer adhesion in connection with the density and type of chemical bonds at the interface. The short-beam strength corresponding to oxygen fractions of 0.61, 0.71 and powers of 5, 10, 15 W is expected to be in the range of 23.1 to 45.2 MPa. The short-beam strength for unsized and commercially sized GFs was 15.1 MPa and 39.2 MPa, respectively. SEM (scanning electron microscopy) micrographs of the damaged composite beams were similar to each other and did not allow us to distinguish between higher and lower short-beam strength, with the exception of unsized fibers. Examples of SEM micrographs can be found in the study by Zvonek et al. [19].

**Figure 3:**
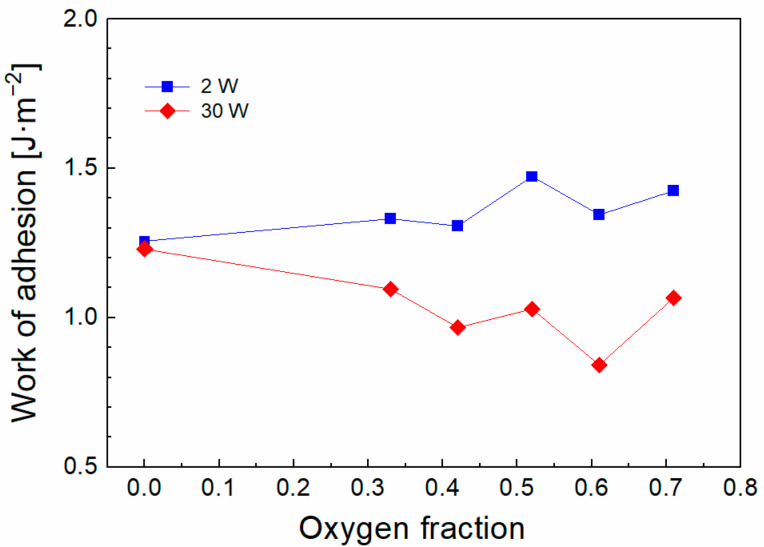
Adhesion of the interlayer deposited at 2 W and 30 W at different oxygen fractions [20].

**Figure 4:**
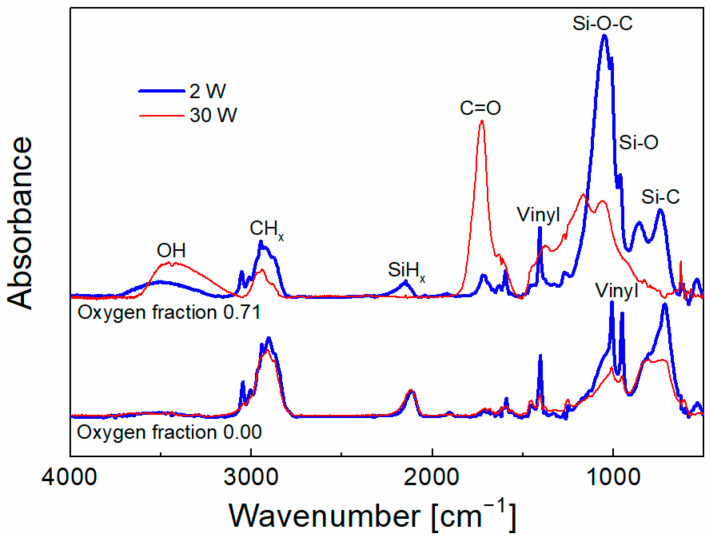
Infrared spectra corresponding to interlayers deposited at 2 W and 30 W and two oxygen fractions, zero and 0.71.

Previous results have shown that the shear strength of the composite is controlled by the adhesion of the interlayer (plasma nanocoating) to the fiber, which is a key factor, and can be characterized by the work of adhesion [20]. The observation of both interfaces in the cross-section of the GF/polyester composite confirmed the essential role of adhesion at the interlayer/fiber interface [16]. The work of adhesion as a function of oxygen fraction for 2 and 30 W is plotted in Figure 3. The level of interlayer adhesion for 2 W increased slightly with increased oxygen fraction and conversely decreased for interlayers deposited at 30 W. The surface of unsized GFs pretreated with oxygen plasma contains hydroxyl groups (Si–OH). Based on the previous chemical analysis of plasma coatings [21] and plasma analysis [25], it can be expected that the silicon-containing plasma species (Si–vinyl_x_, where x = 1–3), characteristic of plasma at lower power (2 W), are responsible at the zero oxygen fraction for higher adhesion due to the formation of stronger Si–O–Si species (369 kJ mol^−1^ [27]) at the interlayer/glass interface, which was confirmed by detailed chemical analysis of this interface, where Si–O and Si–O_2,3_ species dominate [28]. However, plasma at a higher power (30 W) results in an increase in carbon plasma species (C_2_H_x_, where x = 1–3) [25], which will create Si–O–C species with a similar binding energy (351 kJ mol^−1^ [27]) at the interlayer/glass interface, whose concentration increases with increasing RF power at the expense of Si–O–Si bonds, leading to a similar work of adhesion at zero oxygen fraction (Figure 3). The reduced concentration of silicon-containing species incorporated into the coating at higher power (30 W) can be demonstrated by the FTIR spectra (Figure 4), where the reduced area of the absorption band assigned to the Si–C vibrations (716 cm^−1^) is evident for the zero-oxygen fraction.

By increasing the oxygen fraction at a lower power (2 W), the oxygen atoms that impinge on the GF surface will lead to hydrogen abstraction (Si–OH → Si–O^•^) [25], thus creating free bonding sites that allow the formation of a higher bond density at the interlayer/glass interface, leading to better interlayer adhesion (Figure 3) and shear strength (Figure 2). The higher bond density at the interlayer/glass interface also means a higher hydrolytic stability of this interface in the GF/polyester composite [29]. In the case of higher power (30 W), the dominant carbon species are oxidized to form carbonyl groups (C=O, 1725 cm^−1^) (Figure 4), which limit the bond density at the interlayer/glass interface and lead to a decrease in interlayer adhesion (Figure 3) and shear strength (Figure 2) at an increased oxygen fraction. According to the model simulation [21], the shear stress at the interlayer/glass interface due to external mechanical loading increases progressively with the increase in the elastic modulus of the interlayer, which may lead to a decrease in the shear strength of the composite. The interlayer modulus of 4 GPa (zero oxygen fracture) decreased only slightly to 3 GPa with increased oxygen fraction for interlayers deposited at 2 W, while the higher power of 30 W resulted in a higher interlayer modulus of 10 GPa (zero oxygen fracture), which increased up to 28 GPa with increased oxygen fraction [20]. Therefore, the increased interlayer modulus for the 30 W series of composite samples is also unfavorable for the short-beam strength (Figure 2). The shear stress distribution across the interphase region towards the matrix/interlayer interface decreases significantly and is only slightly dependent on the elastic modulus of the interlayer. Therefore, the interfacial shear strength at this interface should be only slightly higher than the shear yield strength of the polymer matrix, as discussed in detail by Palesch et al. [30].

## 4. Conclusions

The surface of unsized GFs was pretreated with oxygen plasma and plasma-coated with TVS mixed with oxygen gas in a single-segment reactor using a nonthermal plasma with a higher degree of dissociation. This modification made it possible to vary the short-beam strength of GF/polyester composites in the widest range from 23.1 to 45.2 MPa by varying the RF power from 2 to 30 W at a higher oxygen fraction (0.61–0.71). However, the short-beam strength may vary to a lesser extent with reduced oxygen content. The goal of developing fiber surface modifications is not the highest possible shear strength of the polymer composite but only sufficient shear strength with respect to optimal properties in tension, bending, and compression (or other functional properties) for the given application of the composite. Therefore, considering the required short-beam strength for a given composite product, it is therefore, possible to choose the correct oxygen fraction and power that must be used for the plasma coating of glass fibers. Both the selected power and the selected oxygen fraction determine the interfacial adhesion between the fiber and the plasma coating (interlayer), which is responsible for the corresponding short-beam strength. The interfacial adhesion expressed by the work of adhesion is related to the density of chemical bonds at this interface. The higher the bond density, the higher the interfacial adhesion and the higher the short-beam strength. The results from the single-segment reactor can be used in a roll-to-roll plasma system [21] enabling continuous surface modification of fibers for commercial fiber processing. An extension of the range of shear strength towards higher values could be brought about by gradient interphase, where the interlayer would continuously change from glass to polyester resin in a chemical and mechanical sense.

## Data Availability

The data presented in this study are available on request from the corresponding author.
